# MicroRNA sequence polymorphisms and the risk of different types of cancer

**DOI:** 10.1038/srep03648

**Published:** 2014-01-13

**Authors:** Ye Hu, Chen-Yang Yu, Ji-Lin Wang, Jian Guan, Hao-Yan Chen, Jing-Yuan Fang

**Affiliations:** 1 Division of Gastroenterology and Hepatology, Renji Hospital, School of Medicine, Shanghai Jiao Tong University, Shanghai Institution of Digestive Disease; Key Laboratory of Gastroenterology & Hepatology, Ministry of Health; State Key Laboratory of Oncogene and Related Genes. 145 Middle Shandong Rd, Shanghai 200001, China; 2 Department of Otolaryngology, The Affiliated Sixth People's Hospital,Otolaryngology Institute of Shanghai Jiao Tong University, Shanghai 200233, China; 3 These authors contributed equally to this work.

## Abstract

MicroRNAs (miRNAs) participate in diverse biological pathways and may act as oncogenes or tumor suppressors. Single nucleotide polymorphisms (SNPs) in miRNAs (MirSNPs) might promote carcinogenesis by affecting miRNA function and/or maturation; however, the association between MirSNPs reported and cancer risk remain inconsistent. Here, we investigated the association between nine common MirSNPs and cancer risk using data from large scale case-control studies. Eight precursor-miRNA (pre-miRNA) SNPs (rs2043556/miR-605, rs3746444/miR-499a/b, rs4919510/miR-608, rs2910164/miR-146a, rs11614913/miR-196a2, rs895819/miR-27a, rs2292832/miR-149, rs6505162/miR-423) and one primary-miRNA (pri-miRNA) SNP (rs1834306/miR-100) were analyzed in 16399 cases and 21779 controls from seven published studies in eight common cancers. With a novel statistic, Cross phenotype meta-analysis (CPMA) of the association of MirSNPs with multiple phenotypes indicated rs2910164 C (*P* = 1.11E-03), rs2043556 C (*P* = 0.0165), rs6505162 C (*P* = 2.05E-03) and rs895819 (*P* = 0.0284) were associated with a significant overall risk of cancer. In conclusion, MirSNPs might affect an individual's susceptibility to various types of cancer.

The worldwide cancer burden continues to increase; however, the precise mechanisms of carcinogenesis remain largely unknown. A number of investigators have demonstrated that genetic factors play a significant role in an individual's risk of cancer. MicroRNAs (miRNAs) are naturally occurring, small, noncoding, single-stranded RNA molecules that regulate gene expression by base pairing with the 3′ untranslated region of their target mRNAs, leading to mRNA cleavage or translational repression^1^. Numerous studies have demonstrated that miRNAs regulate a variety of biological processes, including cell proliferation, differentiation, apoptosis and development, thus dysregulation of these processes is closely associated with carcinogenesis^2^,^3^.

Recently, single nucleotide polymorphisms (SNPs) located in miRNAs, named as MirSNPs, have attracted increasing attention due to their possible involvement in the development of various types of cancer. Such MirSNPs may play functional roles through affecting the transcription of the primary target gene, altering pri-miRNA/pre-miRNA processing, or exerting effects on miRNA-mRNA interactions^4^. We performed a literature search and review of the association of common MirSNPs, including rs1834306, rs2043556, rs3746444, rs4919510, rs2910164, rs11614913, rs895819, rs2292832 and rs6505162, with the risk of cancer. However, the conclusions of the relevant studies were inconsistent, in part because of the heterogeneity of the types of cancer studied, the small sample sizes, and the varied ethnicity of the patients. Therefore, there is an urgent need to further investigate the association of cancer-related MirSNPs with the risk of various types of cancer. Although the identification of cancer-related miRNAs based on gene association studies has become increasingly popular^5^, no study has yet investigated the association of cancer-related MirSNPs with the risk of various types of cancer based on an analysis of a large number of MirSNP association studies.

Therefore, we conducted a candidate-gene designed association study employing large numbers of cases and controls for eight kinds of cancer that commonly jeopardize human health (bladder cancer, breast cancer, esophageal squamous cell carcinoma (ESCC), gastric cancer, lung cancer, pancreatic cancer, and renal cell carcinoma (RCC)), and analyzed these nine MirSNPs (either by direct genotyping or imputation) to further determine the association of these MirSNPs with the risk of developing cancer. Cross phenotype meta-analysis (CPMA) was performed to analyze the association of MirSNPs and overall cancer risk, and specific cancer risk was further discussed.

## Results

### Patient characteristics

The risk of developing eight different types of cancer, including bladder cancer, breast cancer, lung cancer, pancreatic cancer, RCC, prostate cancer, ESCC, and gastric cancer was assessed. The patients and controls in the gastric cancer and ESCC study were from Asian population, while the patients and controls in the six other cancer studies were from a Caucasian population.

### Quantitative analysis

Primary analyses were conducted through unconditional logistic regression models for genotype trend effects (1 degree of freedom) and adjusted for eigenvectors, gender and cohort. The false discovery rate (FDR) method was considered to correct for multiple testing. Results revealed a significant association between rs2910164 C vs G and the risk of bladder cancer (OR = 1.12, 95% CI: 1.04–1.21, *P* = 2.06E-03(*P*_FDR_ = 0.0297)) and gastric cancer (OR = 0.85, 95% CI: 0.77–0.93, *P* = 5.98E-04(*P*_FDR_ = 0.0108)); rs2043556 C vs. T and the risk of bladder cancer (OR = 1.19, 95% CI: 1.10–1.28, *P* = 1.44E-05(*P*_FDR_ = 5.18E-04)); rs6505162 C vs. A and the risk of bladder cancer (OR = 1.12, 95% CI: 1.068–1.18, P = 4.05E-05(PFDR = 9.72E-04)); rs895819 C vs. T and the risk of bladder cancer (OR = 1.19, 95% CI: 1.10–1.28, P = 6.70E-06(PFDR = 4.82E-04)). We further performed the above analyses 1000 times but randomly selected 70% cases and controls each time, and results showed that the five MirSNPs mentioned above were consistently associated with specific type of cancer risk (*P* < 0.001).

**Figure 1:**
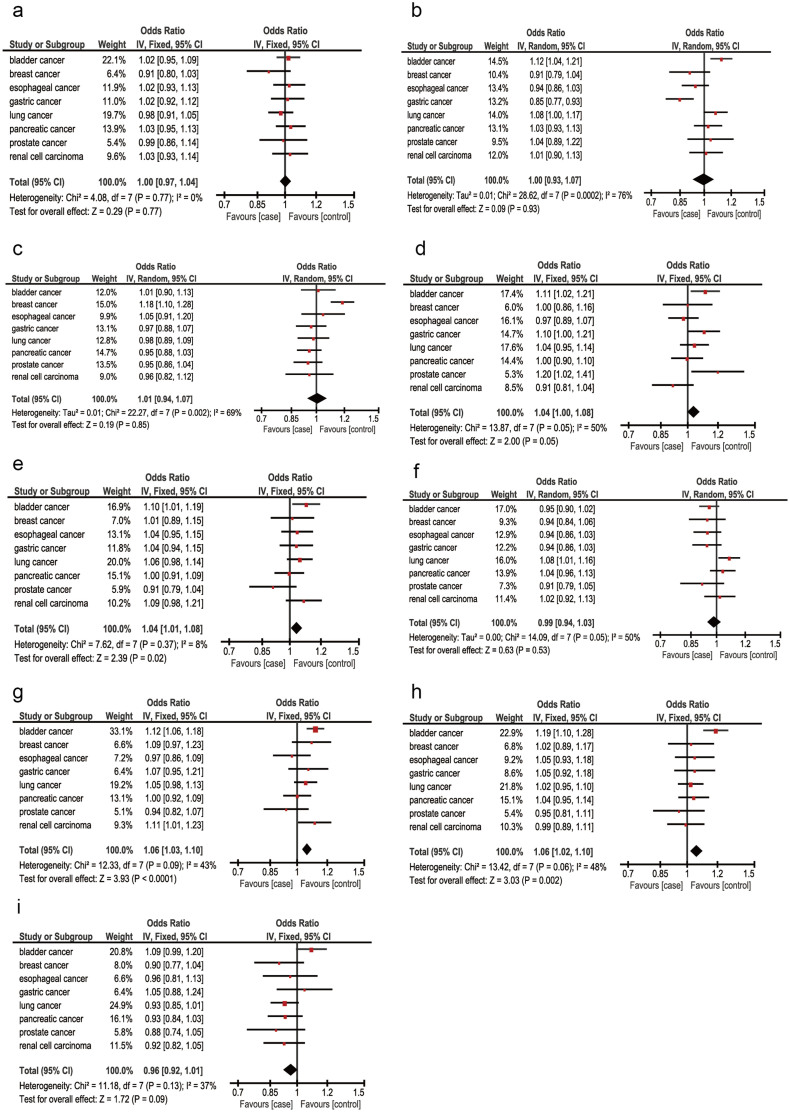
Meta-analysis of nine MirSNPs and their association with the overall risk of cancer. (a) rs2292832; (b) rs2910164; (c) rs2043556; (d) rs4919510; (e) rs1834306; (f) rs1614913; (g) rs6505162; (h) rs895819; (i) rs3746444.

CPMA analysis was performed to unveil the association of each MirSNP with the overall risk of cancer, which suggested that rs2910164 C (*P* = 1.11E-03), rs2043556 C (*P* = 0.0165), rs6505162 C (*P* = 2.05E-03) and rs895819 (*P* = 0.0284) involved with cancer occurrence (Table 2). A meta-analysis using different effects model with inverse-variance weighting based on the heterogeneity existing in the results of the studies in each MirSNP was also provided (Figure 1).

**Figure 2:**
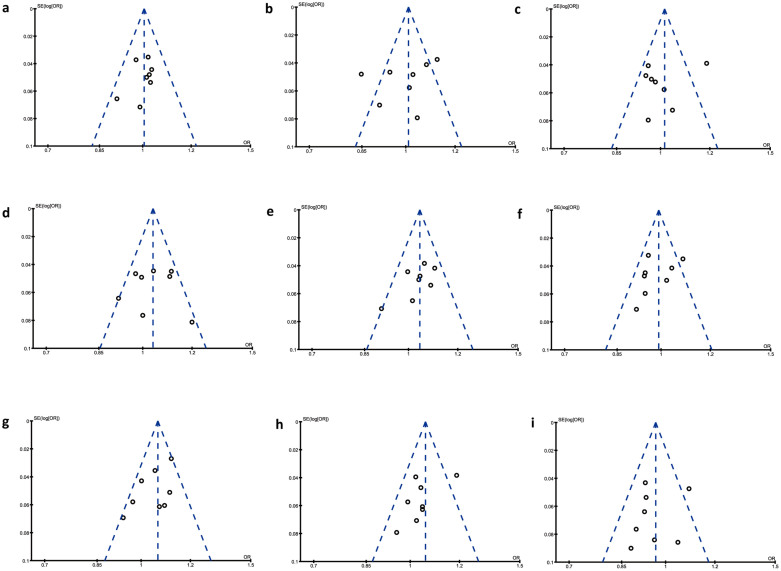
Begg's funnel plot with pseudo 95% confidence limits for publication bias of the MirSNPs in meta-analysis. Begg's test offers no evidence of publication bias. (a) rs2292832 (*P* = 0.386); (b) rs2910164 (*P* = 0.536); (c) rs2043556 (*P* = 0.386); (d) rs4919510 (*P* = 1.000); (e) rs1834306 (*P* = 0.174); (f) rs11614913 (*P* = 0.536); (g) rs6505162 (*P* = 0.174); (h) rs895819 (*P* = 0.386); (i) rs3746444 (*P* = 1.000).

Begg's test was used to investigate publication bias in the literature. The shapes of the funnel plots showed no obvious asymmetry and no statistical evidence of bias existed (Figure 2).

## Discussion

Approximately 50% of all annotated human miRNA genes are located in fragile sites or areas of the genome that are frequently associated with cancer. SNPs, the most common type of genetic variation in the human genome, result in phenotypic differences^6^; such sequence variations in miRNA genes may potentially affect the processing of miRNAs, pri-miRNAs, pre-miRNAs and/or mature miRNAs, and/or target selection and may thus significantly affect an individual's risk of cancer^7^.

Here we evaluated the associations between nine common MirSNPs (rs1834306, rs2043556, rs3746444, rs4919510, rs2910164, rs11614913, rs895819, rs2292832 and rs6505162) and the susceptibility to cancer using data from seven published studies; each study investigated a single type of cancer, except for one study which investigated both gastric adenocarcinoma and ESCC. Therefore, this study was a large population-based and multi-cancer stratified investigation. We observed significant relations between the MirSNPs rs2910164, rs2043556, rs6505162 and the overall risk of developing cancer using FDR adjusted CPMA analysis. CPMA analysis adopts association *P* values and examine whether the observed *P* values diverge from the expected distribution of *P* values under the null hypothesis of no additional associations besides those already known. The CPMA analysis is especially well fitted to wide phenotypic surveys, resulting from its benefits from increased numbers of phenotypes^8^.

The rs2910164 G/C polymorphism of the miR-146a gene is situated in the stem structure opposite the mature miR-146 sequence, and leads to a change from a G:U pair to C:U mismatch in the stem region of the miR-146a precursor. The G allele of the miR-146a precursor might influence the generation of mature miR-146a and impact on target mRNA binding^9^,^10^. Our study revealed an association between rs2910164 and the overall risk of cancer by CPMA, which is inconsistent with He et al using random effects meta-analysis^11^. Although random effects meta-analysis incorporates a moderate level of the effects of heterogeneity, it is not well suited for the cases in which the genetic variant produces the opposite effects on diverse phenotypes. For rs2910164, the results of the two different meta-analyses may be due to the opposite effects of the MirSNP in different types of cancer, thus the use of CPMA seems more reasonable^10^,^11^,^12^,^13^. It is of interest to learn that the amount of mature miR-146a from the C allele were 1.8-fold reduced, compared to the G allele in papillary thyroid carcinoma, while the miR-146a levels in the CC genotype were significantly increased compared with the GG genotype in gastric cancer^13^. The rs2910164 C allele was associated with a decreased risk of gastric cancer in the Asian population, a finding supported by Xu et al^14^. An increased risk of bladder cancer in the Caucasian population was observed in the rs2910164 C allele. However, a study performed by Wang et al. indicated a reduced risk of bladder cancer in the rs2910164 C allele in Asian population^15^. These results suggest that the rs2910164 polymorphism may have varying effects in different genetic backgrounds or patients with a different ethnicity, and/or during the pathogenesis of different types of cancer.

The rs6505162 SNP, located in the pre-miR-423, 12 base pairs 5′ of miR-423-5p offers an association with cancer development based on CPMA analysis. So far, most research on miR-423 has concentrated on expression analyses, where aberrant expression of both mature forms of the miRNA has been seen in cancer, as well as during cellular differentiation^16^,^17^,^18^,^19^. Studies have shown that pre-miRNA SNPs from miRNAs can affect the production of mature forms and the binding of nuclear factors related to miRNA processing^20^,^21^,^22^. We suppose that rs6505162 might affect the expression or processing of miR-423, therefore, studies evaluating the effect of this SNP in miRNA functionality are required. However, studies of the rs6505162 polymorphism on cancer risk have yielded inconsistent results^23^,^24^,^25^. The first of these studies was conducted in 2009 on ESCC in a population of 346 Caucasian ESCC patients and suggested the C allele of rs6505162 being significantly higher in cancer patients compared with controls^23^. A study performed in 2012 indicated that the C genotype of the rs6505162 SNP reduces the risk of breast cancer development, however, another study undertaken in 2009 suggested that the C genotype of rs6505162 offered an increased risk of developing both ovarian and breast cancer in Breast Cancer Associated 2 (BRCA2) mutation carriers^26^. Our research observed an increased risk of bladder cancer in the rs6505162 C allele using the Caucasian population, as to our knowledge, this is the first study to show a relation between this SNP and bladder cancer, thus needs further validation.

The allele C of rs2043556, located in miR-605, was marginally associated with a risk of developing cancer; this is the first study to associate this MirSNP with cancer development, which needs to be validated by more studies. Stratified analysis revealed that the miR-605 allele C was associated with an increased risk of developing bladder cancer in the Caucasian population. Recently, analysis of this SNP was conducted on gastrointestinal cancer among Asians and produced data similar to our own, with C allele being significantly lower in controls compared to cancer patients^27^. Researchers have found that miR-605 to be an element of the p53 network which forms a positive feedback loop in response to stress^28^, thus miR-605 may play a key role in carcinogenesis. It will make more sense if the association between SNP and miRNA expression have been investigated and might be an answer to the relation of SNP and cancer risk.

The allele C of the MirSNP rs895819, located in the terminal loop of the pre-miR-27a, was associated with increased risk of bladder cancer in the Caucasian population, and it is the first study to address association between the MirSNP and bladder cancer. MiR-27a has been investigated in several types of cancer and comes into inconsistent results. MiR-27a functions as a tumor suppressor in ESCC and hepatocellular carcinoma, while serves as promoting factor in gastric tumorigenesis^29^,^30^. Therefore, we assume that miR-27a plays pleiotropic signaling roles in regulating tumorigenesis. The MirSNP rs895819 initially reported to relate with a reduced risk of familial breast cancer risk (*P* = 0.0215) in a Caucasian population^31^; however, no significant association of rs895819 with the risk of breast cancer was observed in Chinese population^32^. A previous study suggested no association between rs895819 and the risk of colorectal cancer in a Central-European Caucasian population, a population with an extremely high incidence of sporadic colorectal cancer^33^; this observation is supported by our results. Since the high probability of MirSNP rs895819 involved with carcinogenesis, these conflicting results may be due to the analysis of varying sample sizes, and warrant further analysis of larger cohorts to clearly establish the impact of rs895819 on the risk of cancer.

The rs11614913 polymorphism of miR-196a2 has a significant impact on the expression of miR-196a2 and is associated with carcinogenesis in various types of cancer^34^,^35^. Previous, meta-analysis studies suggested a significant association between rs11614913 and the overall risk of cancer in the Asian population, which was inconsistent with our results^11^,^36^,^37^. Our study suggests the rs116114913 C allele might protect against lung cancer in the Caucasian population, but the significance was mitigated with *P* value 0.197 after FDR adjustment, while Tian et al found rs116114913 C allele associated with significantly increased risk of lung cancer in Chinese^38^, suggesting that the effect of the rs11614913 polymorphism may rely on the genetic background or ethnicity of the patients and/or the effects of the environment, in agreement with the reports of Chu et al.^37^ and Wang et al.^36^. The effect of rs11614913 on the risk of different types of cancer needs to be confirmed in additional studies.

No significant associations were observed for the rs1834306, rs4919510, rs2292832 and rs3746444 polymorphisms in terms of the overall risk of cancer or the risk of specific types of cancer.

Though miR-100 has been shown to suppress the expression of proteins in the insulin-like growth factor (IGF)/mammalian target of rapamycin (mTOR) signaling cascade in childhood adrenocortical tumors^39^ and clear cell ovarian cancer^40^, thus suppressing tumorigenesis, while act as a oncogene in acute myeloid leukemia^41^. Our results showed mir-100 polymorphism, located in the pri-miR-100 region had no relation with the risk of cancer. Rs4919510 lies within the mature miR-608 sequence, and is located at the junction between the stem and canonical hairpin loop^42^. Rs4919510 G allele was observed to relate with increased risk in bladder cancer, gastric cancer and prostate cancer, however, *P* values were mitigated after FDR adjustment, which needs to be validated by further studies. Rs2292832, located in pre-miR-149, was previously reported to have no significant associations with the risk of evaluated in breast cancer^43^, lung cancer^44^ or gastrointestinal cancer^45^, in agreement with the results of this study. Although a sizeable number of studies have been performed to investigate the role of the miR-499 rs3746444 polymorphism in several types of cancer, including breast cancer^43^,^46^, lung cancer^44^, gastric cancer^47^ and bladder cancer^48^, these existing studies have yielded contradictory results. These discrepancies may be due to the study of different populations from different areas and variations in selection of the case groups; therefore, the effect of the miR-499 rs3746444 polymorphism needs to be investigated further.

One limitation of the present study that needed to be addressed is the multiple comparison problems resulting from the number of MirSNPs tested. Therefore the FDR method was used to correct for multiple testing.

Second, several MirSNPs were imputed rather than directly genotyped in this study. Although using imputed MirSNPs might lead to less accurate results, we ensured that only SNPs with high imputation confidence > 95% were included into further analysis.

Taken together, the findings of the present study have substantial scientific significance and may have implications in the clinical setting. Our results suggest that common MirSNPs may contribute to an individual's susceptibility to diverse types of cancer. Further functional characterization of MirSNPs and their influence on their target mRNAs may reveal the underlying mechanisms responsible for the associations between these polymorphisms and the etiology of cancer. Further prospective investigations of larger numbers of cases and controls are required in order to clarify the inconsistent associations between MirSNPs and the risk of cancer.

## Methods

### Identification of eligible studies

We evaluated the effect of nine MirSNPs on the risk of bladder cancer in 3527 cases and 5119 controls from the Maryland bladder cancer study (dbGAP number: phg0000132.v1) performed among the Caucasian population of the United States^49^; the risk of breast cancer in 1145 postmenopausal women of European ancestry with invasive breast cancer and 1142 controls from the Massachusetts breast cancer study (dbGAP number: phg000032.v1) performed in the United States^50^; the risk of lung cancer in 3782 cases and 3840 controls from the Maryland lung cancer study (dbGAP number: phg000124.v1) performed in the United States^51^; the risk of pancreatic cancer in 2452 affected individuals (cases) and 2461 unaffected controls from the Minnesota pancreatic cancer study (dbGAP number: phg000089.v1) performed in the United States^52^; the risk of prostate cancer in a nested case-control study (dbGAP number: phg000067.v1) including 659 cases and 1593 controls of European origin performed in the United States^53^; the risk of RCC in 1311 affected individuals and 3424 controls with a European background from the Maryland renal cell carcinoma study (dbGAP number: phg000123.v1) performed in the United States^54^; and the risk of gastric adenocarcinoma and ESCC in a study (dbGAP number: phg000128.v1) performed in the United States of individuals of Chinese ethnicity, including 1625 cases of gastric cancer, 1898 cases of ESCC and 2100 controls^55^.

This study is based on an in-silicon re-analyze of the human genotyping data downloaded form dbGAP(www.ncbi.nlm.nih.gov/gap). The data submitters have obtained the informed consent from each participant.

### Selection of SNPs

We carried out a search of the PubMed and Embase databases for all relevant reports on the association of MirSNPs with the risk of cancer. The following candidate MirSNPs were selected for this study: miR-605 A/G (rs2043556), miR-499a/b A/G (rs3746444), miR-608 C/G (rs4919510), miR-146a G/C (rs2910164), miR-196a2 C/T (rs11614913), miRNA-27a T/C (rs895819), miR-149 C/T (rs2292832), miR-423 A/C (rs6505162), and miR-100 T/C (rs1834306), which are present in the pre-miRNA regions of miR-196a2, miR-146a, miR-499a/b, miR-423, miR-608, miRNA-27a, miR-149 and miR-605, and the pri-miRNA region of miR-100, respectively (Table 1).

### Imputation of the MirSNPs

The SNPs not present in the original chip were imputed by the program IMPUTE2, using both HapMap (NCBI Build 36 (db126b)) CEU data and 1000 Genomes as a reference haplotype set. All SNPs showed high imputation confidence (>95%). Rs2292832, rs2043556 and rs11614913 were directly genotyped, the others were imputed.

### Association testing and adjustment for covariates

All the association tests were performed by Plink v1.07 using additive logistic regression models. To account for potential population stratification or admixture in these samples, principal component analyses (PCA) was carried out using the EIGENSTRAT^56^. After adjustment for significant principal components (PCs) in each study based on leveling off of the PCA screen plot, there was no evidence for large scale inflation of the association test statistics by comparison of observed and expected distributions, ruling out the significance hidden population substructure. The principal component score for each individual was included as a covariate in all models along with gender and cohort in logistic regression models. Multivariate logistic regression was performed in R software package (http://www.r-project.org/). The FDR method was used to correct for multiple testing (FDR q < 0.05).

#### Resampling

To examine the robustness of the associations, we conduct a re-sampling analysis in accordance with Li et al.^57^. Using the association test mentioned above, *P*-values (*P*_random_) were obtained by performing the test 1,000 times but randomly selected 70% of population in corresponding study. Then we tested the null hypothesis, *P*_random_ ≥ 0.05(Supplementary Table S1).

### Statistical analysis

The associations of the nine MirSNPs with the risk of cancer were examined by performing meta-analysis using inverse-variance method. We examined the association of the MirSNPs with the overall risk of cancer as measured by odds ratios (ORs) and 95% confidence intervals (CIs). Moreover, stratified analyses were also performed by the type of cancer for each MirSNP. The heterogeneity of the cancer type between studies was evaluated using the Chi-square-based Q statistical test, with a heterogeneity (Ph) < 0.05 considered significant. A fixed-effect model using the Mantel–Haenszel method and a random-effects model using the DerSimonian and Laird method were used to pool the data according to the cancer types and individual MirSNPs. The random-effects model was used when heterogeneity existed in the results of the studies; otherwise the fixed-effect model was used.

Additionally, cross phenotype meta-analysis (CPMA) was performed to determine the associations of the MirSNPs with the overall cancer risk; *P* < 0.05 was considered significant after FDR adjustment. The CPMA statistic determines evidence for the hypothesis that single SNP has multiple phenotypic associations. The CPMA statistic is agnostic to the direction of effect in each disease. It has one degree of freedom as it measures a deviation in *P* value behavior instead of testing all possible combinations of diseases for association to each SNP, and therefore provides high power to reject the null hypothesis^58^,^59^.

All statistical tests for the meta-analysis were performed with review manager version 5.2 (The Nordic Cochrane Centre, The Cochrane Collaboration, Copenhagen, Denmark). Begg's test was used to evaluate publication bias.

## Supplementary Material

Supplementary Information Supplementary Table S1

## Figures and Tables

**Table 1:** Summary of the MirSNPs studied

	Author	Year	Race	Cancer type	Method	Case/control	Polymorphism site	cancer risk
1	Jazdzewski K	2008	Caucasian	Papillary thyroid carcinoma	Northern blot analysis	608/901	rs2910164	yes
2	Tian T	2009	Asian	Lung cancer	PCR-RFLP	1058/1035	rs11614913	yes
3	Hu Z	2009	Asian	Breast cancer	PCR-RFLP	1009/1093	rs2910164,rs2292832,rs11614913,rs3746444	rs11614913,rs3746444 yes, others no risk
4	Li XD	2010	Asian	Hepatocellular carcinoma	PCR-RFLP	310/222	rs11614913	yes
5	Kim MJ	2010	Asian	Lung cancer	Melting-curve analysis	644/644	rs11614913	yes
6	Okubo M	2010	Asian	Gastric cancer	PCR-RFLP	552/697	rs2910164,rs11614913,rs3746444	yes
7	Liu Z	2010	Caucasian	Squamous cell carcinoma of the head and neck	PCR-RFLP	1109/1131	rs2910164,rs2292832,rs11614913,rs3746444	rs3746444 yes, others no risk
8	Catucci I	2010	Caucasian	Breast cancer	Taqman	1897/2760	rs2910164,rs11614913,rs3746444	rs11614913,rs3746444 yes, others no risk
9	Zeng Y	2010	Asian	Gastric cancer	PCR-RFLP	304/304	rs2910164	yes
10	Yang R	2010	Caucasian	Breast cancer	Taqman	1217/1422	rs895819	yes
11	Sun Q	2010	Asian	Gastric cancer	PCR-RFLP	304/304	rs895819	yes
12	Zhan JF	2011	Asian	Colorectal cancer	PCR-RFLP	252/543	rs11614913	yes
13	Farazi TA	2011	Asian	Breast tumors	Small RNA sequencing	168/11	rs6505162	no risk
14	Zhou B	2011	Asian	Cervical squamous cell carcinoma	PCR-RFLP	226/309	rs2910164,rs11614913,rs3746444	rs2910164,rs3746444 yes,others no
15	Akkiz H	2011	Mix	Hepatocellular carcinoma	PCR-RFLP	222/222	rs3746444	no risk
16	Vinci S	2011	Caucasian	Lung cancer	Melting-curve analysis	101/101	rs2910164,rs2292832,rs11614913,rs3746444	rs291064, rs11614913 yes,others no risk
17	Xiang Y	2012	Asian	Primary hepatocellular carcinoma	PCR-RFLP	100/100	rs2910164,rs3746444	yes
18	Chen H	2012	Asian	Colorectal cancer	PCR-LDR	126/407	rs11614913	no risk
19	Smith RA	2012	Caucasian	Breast cancer	Melting-curve analysis	193/193	rs6505162	yes
20	Zhou J	2012	Asian	Primary liver cancer	PCR-RFLP	186/483	rs2910164,rs3746444	no risk
21	Mittal RD	2011	Asian	Bladder cancer	PCR-RFLP	212/250	rs2910164,rs11614913,rs3746444	no risk
22	Ryan BM	2012	Mix	Colorectal cancer	Taqman	245/466	rs4919510	no risk
23	Huang AJ	2012	Asian	Breast tumors	SNPlex assay	1432/1934	rs4919510	yes
24	Shi D	2012	Asian	Renal cell cancer	Taqman	594/600	rs895819	yes
25	Hezova R	2012	Caucasian	Colorectal cancer	TaqMan	192/212	rs11614913,rs895819,rs2910164	no risk
26	Zhou Y	2012	Asian	Gastric cancer	Locus specific single-base extension reactions	311/425	rs895819,rs6505162,rs2910164,rs7372209,rs531564	rs895819 yes, others no risk
27	Zhang M	2012	Asian	Breast cancer	PCR-RFLP	252/248	rs2043556,rs2292832,rs895819,rs11614913,rs2682818	no risk
28	Zhang MW	2012	Asian	Gastrointestinal cancer	PCR-RFLP	762/757	rs2043556,rs2292832	no risk
29	Wei WJ	2013	Asian	Thyroid carcinoma	iPLEX GOLD	753/1244	rs2910164	no risk
30	Ma L	2013	Asian	Colorectal cancer	TaqMan	1147/1203	rs2910164	yes
31	Orsós Z	2013	Caucasian	Head and neck cancer	PCR-CTPP	468/468	rs2910164	yes
32	Yamashita J	2013	Asian	Malignant melanoma	PCR-RFLP	50/107	rs2910164	yes

**Table 2:** Stratification analyses of the association of the nine MirSNPs with the overall cancer risk and risk of specific types of cancer

				Bladder cancer	Breast cancer	ESCC***	Gastric cancer	Lung cancer	Pancreatic cancer	Prostate cancer	RCC***	
				Case/Control								
SNP				3527/5119	1145/1142	1898/2100	1625/2100	3782/3840	2452/2461	659/1593	1311/3424	
SNP_ID	Chr	MicroRNA	Ref_Allele	*P* value(*P*_FDR_**);OR(95%CI)								*P_CPMA_*(P_FDR_**)
rs2292832*	2	miR149	T	0.5868	0.1418	0.6097	0.7599	0.4977	0.4548	0.8873	0.5841	0.2965
				1.02(0.95–1.09)	0.91(0.80–1.03)	1.02(0.93–1.13)	1.02(0.92–1.12)	0.98(0.91–1.05)	1.03(0.95–1.13)	0.99(0.86–1.14)	1.03(0.93–1.14)	
rs2910164	5	miR146a	C	2.06E-03(0.0297)	0.1667	0.2022	5.98E-04(0.0108)	0.0623	0.5733	0.5841	0.8238	1.11E-03(9.22E-03)
				1.12(1.04–1.21)	0.91(0.79–1.04)	0.94(0.86–1.03)	0.85(0.77–0.93)	1.08(1.00–1.17)	1.03(0.94–1.13)	1.04(0.89–1.22)	1.01(0.90–1.13)	
rs2043556*	10	miR605	C	1.44E-05(5.18E-04)	0.5423	0.4995	0.7126	0.2572	0.2566	0.5694	0.4774	0.0165(0.0495)
				1.19(1.10–1.28)	1.05(0.91–1.20)	0.97(0.88–1.07)	0.98(0.89–1.09)	0.95(0.88–1.03)	0.95(0.86–1.04)	0.96(0.82–1.12)	0.96(0.85–1.08)	
rs4919510	10	miR608	G	0.0182(0.1971)	0.9956	0.5794	0.0418(0.2736)	0.3599	0.9397	0.0260(0.2080)	0.1597	0.0690
				1.11(1.02–1.21)	1.00(0.86–1.16)	0.97(0.89–1.07)	1.10(1.00–1.21)	1.04(0.95–1.14)	1.00(0.90–1.10)	1.20(1.02–1.41)	0.91(0.81–1.04)	
rs1834306	11	miR100	A	0.0213(0.1971)	0.8174	0.3610	0.4405	0.1295	0.9386	0.1609	0.1268	0.2303
				1.10(1.01–1.20)	1.02(0.89–1.15)	1.04(0.95–1.15)	1.04(0.94–1.15)	1.06(0.98–1.14)	1.00(0.91–1.09)	0.91(0.79–1.04)	1.09(0.98–1.21)	
rs11614913*	12	miR196a2	T	0.1500	0.3188	0.1942	0.1970	0.0219(0.1971)	0.3503	0.1988	0.6741	0.1233
				0.95(0.90–1.02)	0.94(0.84–1.06)	0.94(0.86–1.03)	0.94(0.86–1.03)	1.08(1.01–1.16)	1.04(0.96–1.13)	0.91(0.79–1.05)	1.02(0.93–1.13)	
rs6505162	17	miR423	C	4.05E-05(9.72E-04)	0.1494	0.5894	0.2689	0.1480	0.9894	0.3396	0.0376(0.6665)	2.05E-03(9.22E-03)
				1.12(1.06–1.18)	1.09(0.97–1.23)	0.97(0.87–1.09)	1.07(0.95–1.21)	1.05(0.98–1.13)	1.00(0.92–1.09)	0.94(0.82–1.07)	1.11(1.01–1.23)	
rs895819	19	miR27a	C	6.70E-06(4.82E-04)	0.7482	0.4560	0.4794	0.6004	0.4106	0.5272	0.8790	0.0284(0.0639)
				1.19(1.10–1.28)	1.02(0.89–1.18)	1.05(0.93–1.18)	1.05(0.92–1.18)	1.02(0.94–1.10)	1.04(0.95–1.14)	0.95(0.81–1.11)	0.99(0.89–1.11)	
rs3746444	20	miR499a/b	G	0.0684	0.1558	0.6197	0.6012	0.0846	0.1794	0.1575	0.2190	0.1296
				1.09(0.99–1.20)	0.90(0.77–1.04)	0.96(0.81–1.13)	1.05(0.88–1.24)	0.93(0.85–1.01)	0.93(0.84–1.03)	0.88(0.74–1.05)	0.92(0.82–1.05)	

*These SNPs were directly genotyped,the others were imputed.

**The false discovery rate (FDR) method was used to correct for multiple testing (FDR q < 0.05).

***ESCC abbrevaition of esophageal squamous cell carcinoma, RCC abbrevaition of renal cell carcinoma.

## References

[b1] Lagos-Quintana M., Rauhut R., Lendeckel W. & Tuschl T. Identification of novel genes coding for small expressed RNAs. Science. 294, 853–8 (2001). 11679670 10.1126/science.1064921

[b2] Ambros V. MicroRNA pathways in flies and worms: growth, death, fat, stress, and timing. Cell. 113, 673–6 (2003). 12809598 10.1016/s0092-8674(03)00428-8

[b3] Lu J. *et al.* MicroRNA expression profiles classify human cancers. Nature. 435, 834–8 (2005). 15944708 10.1038/nature03702

[b4] Ryan B. M., Robles A. I. & Harris C. C. Genetic variation in microRNA networks: the implications for cancer research. Nat Rev Cancer. 10, 389–402 (2010). 20495573 10.1038/nrc2867 PMC2950312

[b5] Ma Y. *et al.* Candidate microRNA biomarkers in human colorectalcancer: systematicreview profiling studies andexperimental validation. Int J Cancer. 130, 2077–87 (2012). 21671476 10.1002/ijc.26232

[b6] Hinds D. A. *et al.* Whole-genome patterns of common DNA variation in three human populations. Science. 307, 1072–9 (2005). 15718463 10.1126/science.1105436

[b7] Duan R., Pak C. & Jin P. Single nucleotide polymorphism associated with mature miR-125a alters the processing of pri-miRNA. Hum Mol Genet. 16, 1124–31 (2007). 17400653 10.1093/hmg/ddm062

[b8] Solovieff N., Cotsapas C., Lee P. H., Purcell S. M. & Smoller J. W. Pleiotropy in complex traits: challenges and strategies. Nat Rev Genet. 14, 483–95, 10.1038/nrg3461 (2013). 23752797 PMC4104202

[b9] Xu T. *et al.* A functional polymorphism in the miR-146a gene is associated with the risk for hepatocellular carcinoma. Carcinogenesis. 29, 2126–31 (2008). 18711148 10.1093/carcin/bgn195

[b10] Jazdzewski K. *et al.* Common SNP in pre-miR-146a decreases mature miR expression and predisposes to papillary thyroid carcinoma. Proc Natl Acad Sci USA. 105, 7269–74 (2008). 18474871 10.1073/pnas.0802682105 PMC2438239

[b11] He B. *et al.* The association between four genetic variants in microRNAs (rs11614913, rs2910164, rs3746444, rs2292832) and cancer risk: evidence from published studies. PLoS One. 7, e49032 (2012). 23155448 10.1371/journal.pone.0049032 PMC3498348

[b12] Shen J. *et al.* A functional polymorphism in the miR-146a gene and age of familial breast/ovarian cancer diagnosis. Carcinogenesis. 29, 1963–6 (2008). 18660546 10.1093/carcin/bgn172

[b13] Kogo R., Mimori K., Tanaka F., Komune S. & Mori M. Clinical significance of miR-146a in gastric cancer cases. Clin Cancer Res. 17, 4277–84 (2011). 21632853 10.1158/1078-0432.CCR-10-2866

[b14] Xu W. *et al.* Effects of common polymorphisms rs11614913 in miR-196a2 and rs2910164 in miR-146a on cancer susceptibility: a meta-analysis. PLoS One. 6, e20471 (2011). 21637771 10.1371/journal.pone.0020471 PMC3102728

[b15] Wang M. *et al.* Genetic variants in miRNAs predict bladder cancer risk and recurrence. Cancer Res. 72, 6173–82 (2012). 22846912 10.1158/0008-5472.CAN-12-0688

[b16] Kasashima K., Nakamura Y. &Kozu T. Altered expression profiles of microRNAs during TPA-induced differentiation of HL-60 cells. Biochem Biophys Res Commun. 322, 403–410 (2004). 15325244 10.1016/j.bbrc.2004.07.130

[b17] Guled M. *et al.* CDKN2A, NF2, and JUN are dysregulated among other genes by miRNAs in malignant mesothelioma – A miRNA microarray analysis. Genes Chromosomes Cancer. 48, 615–623 (2009). 19396864 10.1002/gcc.20669

[b18] Hui A. B. *et al.* Comprehensive microRNA profiling for head and neck squamous cell carcinomas. Clin Cancer Res. 16, 1129–1139 (2010). 20145181 10.1158/1078-0432.CCR-09-2166

[b19] Farazi T. A. *et al.* MicroRNA sequence and expression analysis in breast tumors by deep sequencing. Cancer Res. 71, 4443–4453 (2011). 21586611 10.1158/0008-5472.CAN-11-0608 PMC3129492

[b20] Xu B. *et al.* A functional polymorphism in Pre-miR-146a gene is associated with prostate cancer risk and mature miR-146a expression in vivo. Prostate. 70, 467–472 (2010). 19902466 10.1002/pros.21080

[b21] Hu Y. *et al.* Two common SNPs in pri-miR-125a alter the mature miRNA expression and associate with recurrent pregnancy loss in a Han-Chinese population. RNA Biol. 8, 861–72 (2011). 21788734 10.4161/rna.8.5.16034

[b22] Akkız H., Bayram S., Bekar A., Akgöllü E. & Ulger Y. A functional polymorphism in pre-microRNA-196a-2 contributes to the susceptibility of hepatocellular carcinoma in a Turkish population: a case-control study. J Viral Hepat. 18, e399–407 (2011). 21692953 10.1111/j.1365-2893.2010.01414.x

[b23] Ye Y. *et al.* Genetic variations in microRNA-related genes are novel susceptibility loci for esophageal cancer risk. Cancer Prev Res. 1, 460–9 (2008). 10.1158/1940-6207.CAPR-08-0135 PMC2768267 19138993

[b24] Farazi T. A. *et al.* MicroRNA sequence and expression analysis in breast tumors by deep sequencing. Cancer Res. 71, 4443–53 (2011). 21586611 10.1158/0008-5472.CAN-11-0608 PMC3129492

[b25] Smith R. A. *et al.* A genetic variant located in miR-423 is associated with reduced breast cancer risk. Cancer Genomics Proteomics. 9, 115–8 (2012). 22593246

[b26] Kontorovich T., Levy A., Korostishevsky M., Nir U. & Friedman E. Single nucleotide polymorphisms in miRNA binding sites and miRNA genes as breast/ovarian cancer risk modifiers in Jewish high-risk women. Int J Cancer. 127, 589–97 (2009). 10.1002/ijc.25065 19950226

[b27] Zhang M. W. *et al.* Associations of lifestyle-related factors, hsa-miR-149 and hsa-miR-605 gene polymorphisms with gastrointestinal cancer risk. Mol Carcinog. 51, E21–31 (2012). 21976437 10.1002/mc.20863

[b28] Xiao J., Lin H., Luo X. & Wang Z. MiR-605 joins p53 network to form a p53: miR-605:Mdm2 positive feedback loop in response to stress. EMBO J. 30, 524–32 (2011). 21217645 10.1038/emboj.2010.347 PMC3034018

[b29] Zhu L., Wang Z., Fan Q., Wang R. &Sun Y. MicroRNA-27a functions as a tumor suppressor in esophageal squamous cell carcinoma by targeting KRAS. Oncol Rep. 31, 280–6 (2013). 24154848 10.3892/or.2013.2807

[b30] Chen Z. *et al.* MiR-27a modulates the MDR1/P-glycoprotein expression by inhibiting FZD7/β-catenin pathway in hepatocellular carcinoma cells. Cell Signal. 25, 2693–701 (2013). 24018051 10.1016/j.cellsig.2013.08.032

[b31] Yang R. *et al.* A genetic variant inthe pre-miR-27a oncogene is associated witha reduced familial breast cancer risk. Breast Cancer Res Treat. 121, 693–702 (2010). 19921425 10.1007/s10549-009-0633-5

[b32] Zhang M. *et al.* Associations of miRNA polymorphisms and female physiological characteristics with breast cancer risk in Chinese population. Eur J Cancer Care. 2, 274–80 (2012). 10.1111/j.1365-2354.2011.01308.x 22074121

[b33] Hezova R. *et al.* Evaluation of SNPs in miR-196-a2, miR-27a and miR-146a as risk factors of colorectal cancer. World J Gastroenterol. 18, 2827–31 (2012). 22719192 10.3748/wjg.v18.i22.2827 PMC3374987

[b34] Zhan J. F. *et al.* A functional variant in microRNA-196a2 is associated with susceptibility of colorectal cancer in a Chinese population. Arch Med Res. 42, 144–8 (2011). 21565628 10.1016/j.arcmed.2011.04.001

[b35] Li X. D., Li Z. G., Song X. X. & Liu C. F. A variant in microRNA-196a2 is associated with susceptibility to hepatocellular carcinoma in Chinese patients with cirrhosis. Pathology. 42, 669–73 (2010). 21080878 10.3109/00313025.2010.522175

[b36] Wang F. *et al.* A genetic variant in microRNA-196a2 is associated with increased cancer risk: a meta-analysis. Mol Biol Rep. 39, 269–75 (2012). 21625865 10.1007/s11033-011-0735-0

[b37] Chu H. *et al.* Hsa-miR-196a2 Rs11614913 polymorphism contributes to cancer susceptibility: evidence from 15 case-control studies. PLoS One. 6, e18108 (2011). 21483822 10.1371/journal.pone.0018108 PMC3069063

[b38] Tian T. *et al.* A functional genetic variant in microRNA-196a2 is associated with increased susceptibility of lung cancer in Chinese. Cancer Epidemiol Biomarkers Prev. 18, 1183–7 (2009). 19293314 10.1158/1055-9965.EPI-08-0814

[b39] Doghman M. *et al.* Regulation of insulin-like growth factor-mammalian target of rapamycin signaling by microRNA in childhood adrenocortical tumors. Cancer Res. 70, 4666–75 (2010). 20484036 10.1158/0008-5472.CAN-09-3970 PMC2880211

[b40] Nagaraja A. K. *et al.* A link between mir-100 and FRAP1/mTOR in clear cell ovarian cancer. Mol Endocrinol. 24, 447–63 (2010). 20081105 10.1210/me.2009-0295 PMC2817607

[b41] Zheng Y. S. *et al.* MiR-100 regulates cell differentiation and survival by targeting RBSP3, a phosphatase-like tumor suppressor in acute myeloid leukemia. Oncogene. 31, 80–92 (2012). 21643017 10.1038/onc.2011.208 PMC3253429

[b42] Ryan B. M. *et al.* Rs4919510 in hsa-mir-608 is associated with outcome but not risk of colorectal cancer. PLoS One. 7, e36306 (2012). 22606253 10.1371/journal.pone.0036306 PMC3350523

[b43] Hu Z. *et al.* Common genetic variants in pre-microRNAs were associated with increased risk of breast cancer in Chinese women. Hum Mutat. 30, 79–84 (2009). 18634034 10.1002/humu.20837

[b44] Vinci S. *et al.* Genetic variants in miR-146a, miR-149, miR-196a2, miR-499 and their influence on relative expression in lung cancers. Clin Chem Lab Med. 49, 2073–80 (2011). 21902575 10.1515/CCLM.2011.708

[b45] Zhang M. W. *et al.* Associations of lifestyle-related factors, hsa-miR-149 and hsa-miR-605 gene polymorphisms with gastrointestinal cancer risk. Mol Carcinog. 51, E21–31 (2012). 21976437 10.1002/mc.20863

[b46] Catucci I. *et al.* Evaluation of SNPs in miR-146a, miR196a2 and miR-499 as low-penetrance alleles in German and Italian familial breast cancer cases. Hum Mutat. 31, E1052–7 (2010). 19847796 10.1002/humu.21141

[b47] Okubo M. *et al.* Association between common genetic variants in pre-microRNAs and gastric cancer risk in Japanese population. Helicobacter. 15, 524–31 (2010). 21073609 10.1111/j.1523-5378.2010.00806.x

[b48] Okubo M. *et al.* Investigative role of pre-microRNAs in bladder cancer patients: a case-control study in North India. DNA Cell Biol. 30, 401–6 (2011). 21345130 10.1089/dna.2010.1159

[b49] Rothman N. *et al.* A multi-stage genome-wide association study of bladder cancer identifies multiply susceptibility loci. Nat Genet. 42, 978–84 (2010). 20972438 10.1038/ng.687 PMC3049891

[b50] Hunter D. J. *et al.* A genome-wide association study identifies alleles in FGFR2 associated with risk of sporadic postmenopausal breast cancer. Nat Genet. 39, 870–4 (2007). 17529973 10.1038/ng2075 PMC3493132

[b51] Landi M. T. *et al.* A genome-wide association study of lung cancer identifies a region of chromosome 5p15 associated with risk for adenocarcinoma. Am J Hum Genet. 85, 679–91 (2009). 19836008 10.1016/j.ajhg.2009.09.012 PMC2775843

[b52] Petersen G. M. *et al.* A genome-wide association study identifies pancreatic cancer susceptibility loci on chromosomes 13q22.1, 1q32.1 and 5p15.33. Nat Genet. 42, 224–8 (2010). 20101243 10.1038/ng.522 PMC2853179

[b53] Yeager M. *et al.* Genome-wide association study of prostate cancer identifies a second risk locus at 8q24. Nat Genet. 39, 645–9 (2007). 17401363 10.1038/ng2022

[b54] Purdue M. P. *et al.* Genome-wide association study of renal cell carcinoma identifies two susceptibility loci on 2p21 and 11q13.3. Nat Genet. 43, 60–5 (2011). 21131975 10.1038/ng.723 PMC3049257

[b55] Abnet C. C. *et al.* A shared susceptibility locus in PLCE1 at 10q23 for gastric adenocarcinoma and esophageal squamous cell carcinoma. Nat Genet. 42, 764–7 (2010). 20729852 10.1038/ng.649 PMC2947317

[b56] Price A. L. *et al.* Principal components analysis corrects for stratification in genome-wide association studies. Nat Genet. 38, 904–909 (2006). 16862161 10.1038/ng1847

[b57] Li J. *et al.* Identification of high-quality cancer prognostic markers and metastasis network modules. Nat Commun. 1, 1–8; 10.1038/ncomms1033 (2010). 20975711 PMC2972666

[b58] Evangelou E. & Ioannidis J. P. Meta-analysis methods for genome-wide association studies and beyond. Nat Rev Genet. 14, 379–89 (2013). 23657481 10.1038/nrg3472

[b59] Cotsapas C. *et al.* Pervasive sharing of genetic effects in autoimmune disease. PLoS Genet. 7, e1002254 (2011). 21852963 10.1371/journal.pgen.1002254 PMC3154137

