# Supplementary material for: MicroRNA sequence polymorphisms and the risk of different types of cancer
Source: Sci Rep. 2014 Jan 13;4:3648. doi: 10.1038/srep03648 (PMC5379157; doi:10.1038/srep03648)
Supplement: Supplementary Information — Supplementary Table S1 [file srep03648-s1.pdf]

MicroRNA sequence polymorphisms and the risk of different types of cancer

Ye Hu, Chen-Yang Yu, Ji-Lin Wang, Jian Guan, Hao-Yan Chen, Jing-Yuan Fang

Supplementary Table S1. Resampling analysis of five significant associations

SNP ID	Cancer Type	Number(P<0.05)	Number($P \geqslant$0.05)
rs2910164	bladder cancer	1000	0
rs2043556	bladder cancer	1000	0
rs6505162	bladder cancer	998	2
rs895819	bladder cancer	1000	0
rs2910164	gastric cancer	846	154

The five significant associations were test 1,000 times but randomly selected 70% of population in corresponding study and 1000 *P* values were obtained as showed above.
